# Simple and Reliable Determination of Intravoxel Incoherent Motion Parameters for the Differential Diagnosis of Head and Neck Tumors

**DOI:** 10.1371/journal.pone.0112866

**Published:** 2014-11-17

**Authors:** Miho Sasaki, Misa Sumi, Sato Eida, Ikuo Katayama, Yuka Hotokezaka, Takashi Nakamura

**Affiliations:** Department of Radiology and Cancer Biology, Nagasaki University School of Dentistry, Nagasaki, Japan; Aarhus University, Denmark

## Abstract

Intravoxel incoherent motion (IVIM) imaging can characterize diffusion and perfusion of normal and diseased tissues, and IVIM parameters are authentically determined by using cumbersome least-squares method. We evaluated a simple technique for the determination of IVIM parameters using geometric analysis of the multiexponential signal decay curve as an alternative to the least-squares method for the diagnosis of head and neck tumors. Pure diffusion coefficients (D), microvascular volume fraction (f), perfusion-related incoherent microcirculation (D*), and perfusion parameter that is heavily weighted towards extravascular space (P) were determined geometrically (Geo D, Geo f, and Geo P) or by least-squares method (Fit D, Fit f, and Fit D*) in normal structures and 105 head and neck tumors. The IVIM parameters were compared for their levels and diagnostic abilities between the 2 techniques. The IVIM parameters were not able to determine in 14 tumors with the least-squares method alone and in 4 tumors with the geometric and least-squares methods. The geometric IVIM values were significantly different (p<0.001) from Fit values (+2±4% and −7±24% for D and f values, respectively). Geo D and Fit D differentiated between lymphomas and SCCs with similar efficacy (78% and 80% accuracy, respectively). Stepwise approaches using combinations of Geo D and Geo P, Geo D and Geo f, or Fit D and Fit D* differentiated between pleomorphic adenomas, Warthin tumors, and malignant salivary gland tumors with the same efficacy (91% accuracy = 21/23). However, a stepwise differentiation using Fit D and Fit f was less effective (83% accuracy = 19/23). Considering cumbersome procedures with the least squares method compared with the geometric method, we concluded that the geometric determination of IVIM parameters can be an alternative to least-squares method in the diagnosis of head and neck tumors.

## Introduction

Diffusion occurs because of the non-ending movement of every single molecule [1]. Brown first observed this phenomenon (although Ingenhousz found the phenomenon earlier than Brown did), and Einstein later gave this phenomenon a sound mathematical description considering a free diffusion process, where the molecules only collide with other molecules in a homogeneous container without boundaries. Diffusion-weighted imaging (DWI) is based on MR signal attenuations caused by the displacement of intracellular and extracellular water molecules for a given time. In biological tissues, however, the environment of water molecules can hardly be called homogeneous: membranes, macromolecules, and fibers hamper the diffusion process [2]. Furthermore, there is other incoherent motion within a voxel that can lead to signal attenuation; in particular, the water molecules in blood capillaries exhibit a pseudorandom motion in the tortuous capillaries.

Le Bihan proposed that intravoxel incoherent motion (IVIM) imaging can distinguish between the pure molecular diffusion and motion of water molecules in the capillary network with a single DWI acquisition technique, provided that high b-values (≥200 s/mm^2^) and low b-values (<200 s/mm^2^) are used [3]. The IVIM imaging can be characterized by 3 parameters: pure diffusion coefficient (D); microvascular volume fraction (f); and perfusion-related incoherent microcirculation (D*) [4]. To determine the IVIM parameters from a multiexponential signal decay curve, the least-squares method is usually used [4], [5]. However, the method is cumbersome, and thus may not be suitable for routine clinical use.

Recently, some researchers have applied simplified methods for determining IVIM parameters to characterize tumors in the liver, prostate, and head and neck region [6]–[9]. For example, Lewin et al shoed that the perfusion fraction parameter f determined by using a geometric analysis of DW MR images can be a marker of sorafenib treatment of patients with advanced hepatocellular carcinoma [7]. However, they did not indicate how precise the geometric determination of the perfusion parameter compared with the conventional technique. In addition, Mazaheri et al noted that the linear fit of the logarithmic signal using limited numbers of b-value is statistically less appropriate than fitting the signals to exponential functions using a least-squares method [8]. The authors also suggested the importance of b-value selection used for the simplified IVIM analysis. In simplified methods, the IVIM parameters are estimated by using a limited number [3]–[4] of b-values compared with the authentic IVIM imaging, which uses 9–13 b-values [4], [5], [10], [11]. However, the reliability in measurements and effectiveness in diagnosing tumors with simplified IVIM techniques using limited numbers of b-value has not been fully investigated. Sasaki et al. reported the reproducibility of IVIM parameter measurements in evaluating the technique for functional assessment of the masticator muscles [12]. However, there was no published report that presented the reproducibility of IVIM parameters in diagnosing tumors. In the present study, we directly compared the IVIM parameter values that were determined by a simplified geometric method with those determined by the conventional least-squares method. We have also compared the diagnostic accuracy for diagnosing head and neck squamous cell carcinomas (SCCs) and lymphomas as well as benign and malignant salivary gland tumors between the 2 methods.

## Materials and Methods

### Ethics statement

The Ethics Committee of Nagasaki University approved this study. Informed consent was waived due to the retrospective nature of the study. Patient records/information was anonymized and de-identified prior to analysis.

### Patients

We retrospectively studied DW MR images of patients with head and neck tumors who underwent preoperative MR examinations between March 2003 to April 2012. We selected head and neck tumors from patients (1) who underwent diffusion-weighted MR imaging as well as conventional contrast-enhanced and non-enhanced T1-weighted and fat-suppressed T2-weighted MR imaging; (2) whose tumors were excised and histologically proven; and (3) whose DW images were good in quality without any severe susceptibility artifacts that would interfere with IVIM analysis. Consequently, the study cohort included 105 head and neck tumors (35 benign and 70 malignant tumors) that arose in 94 patients (56 men and 38 women; average age, 62±15 years; age range, 3–91 years). Detailed tumor pathology is listed in Table 1.

**Table 1:** 105 head and neck tumors.

Tumor	n
Benign	35
Salivary gland tumor	
Pleomorphic adenoma	15
Warthin tumor	8
Odontogenic tumor	
Ameloblastoma	2
Keratcysticodontogenic tumor	2
Odontogenic fibroma	2
Odontogenicmyxoma	1
Hemangioma	1
Angiomyoma	1
Myofibroma	1
Papiloma	1
Adenomatous goiter	1
Malignant	70
SCC	25
SCC node	12
Lymphoma	14
Salivary gland tumor	
Carcinoma ex. pleomorphic adenoma	2
Adenoid cystic carcinoma	1
Acinic cell carcinoma	1
Adenocarcinoma	1
Dedifferentiated carcinoma	1
Salivary duct carcinoma	1
Lymph node metastasis from malignant salivary gland tumor	4
Malignant melanoma	1
Nasopharyngeal carcinoma	1
Neuroendocrine carcinoma	1
Papillary thyroid carcinoma	1
Lymph node metastasis from papillary thyroid carcinoma	3
Ameloblastic carcinoma	1
Total	105

SCC, squamous cell carcinoma.

Of 105 tumors, 18 were excluded from the study owing to measurement errors, including 6 pleomorphic adenomas, 2 lymphomas, 2 SCCs (oropharynx and hypopharynx), 1 SCC node, 1 adenocarcinoma, 1 metastatic node from papillary thyroid carcinoma, 1 neuroendocrine carcinoma, 1 ameloblastic carcinoma, 1 hemangioma, 1 keratocystic odontogenic tumor, 1 myxoma.

DW MR images of the healthy parotid glands (n = 21) and the masseter muscles (n = 21) of the contralateral sides in patients with parotid tumor were also analyzed for comparing the IVIM parameters determined by using geometric or least-squares methods.

### MR imaging

MR imaging was performed using a 1.5-T MR unit (Gyroscan Intera 1.5T Master; Philips Healthcare, Best, The Netherlands). 73 patients were scanned by using a 2-channel 17-cm×14-cm (Synergy-Flex M), 7 patients by using a 2-channel 20-cm (Synergy-Flex L) surface coil, and 14 patients by using a 3-channel head and neck coil (Synergy Head Neck).

### T1- and T2-weighted MR imaging

We obtained axial T1- and fat-suppressed (spectral attenuated with inversion recovery, [SPAIR]) T2-weighted MR images (TR/TE/number of signal acquisitions = 500 ms/15 ms/2 and 6385 ms/80 ms/2, respectively) by using a turbo spin-echo (TSE) sequence (TSE factor = 3 and 15, respectively). We used a 200-mm FOV, 256×204 scan and 512×512 reconstruction matrix sizes, a 4-mm slice thickness and a 0.4-mm slice gap. For contrast-enhanced T1-weighted MR imaging, a gadolinium-based agent (gadopentatate dimeglutimine, Magnevist; Bayer Healthcare, Berlin, Germany) was intravenously injected at a dose of 0.2 mL per kg of body weight and a rate of 1.5 mL/s.

### DW MR imaging

Axial DW images (TR/TE = 1625 ms/81 ms) were obtained using single-shot, spin-echo echo planar imaging (SE-EPI). The EPI factor was 47, and Sensitivity Encoding (SENSE) factor was 2. We used a 200-mm FOV, 4-mm slice thickness, 0.4-mm slice gap, and 112×90 matrix size. The measured pixel size was 1.79/2.28/4 mm. We used 11 b-values (0, 10, 20, 30, 50, 80, 100, 200, 300, 400, and 800 s/mm^2^). The total acquisition time was 1 min 53 s per 5 slices.

### Regions of interest

A region of interest (ROI) was manually placed onto each tumor area such that it encompassed as much of the tumor area as possible. The mean ROI area was 3.4±2.8 cm^2^ (0.8–18.1 cm^2^). Visually large cystic or necrotic areas were excluded from the present analysis. We used the contrast-enhanced T1-weighted and fat-suppressed T2-weighted MR images as references to determine tumor areas on the corresponding DW images. We compared the IVIM values between geometric and least-squares methods based on the IVIM values calculated from ROI-averaged signal intensities. We used DW image slices including the 1–3 maximal tumor areas, and the IVIM values obtained from each ROIs were averaged. For the healthy parotid glands and masseter muscles, irregular ROIs were placed so that they included as much of the gland or muscle area as possible, but did not include large vessels, such as the retromandibular vein, or intraglandular main ducts. A radiologist with 20-year experience in head and neck radiology placed ROIs and analyzed IVIM images.

### IVIM analysis based on least squares method

**Figure 1:**
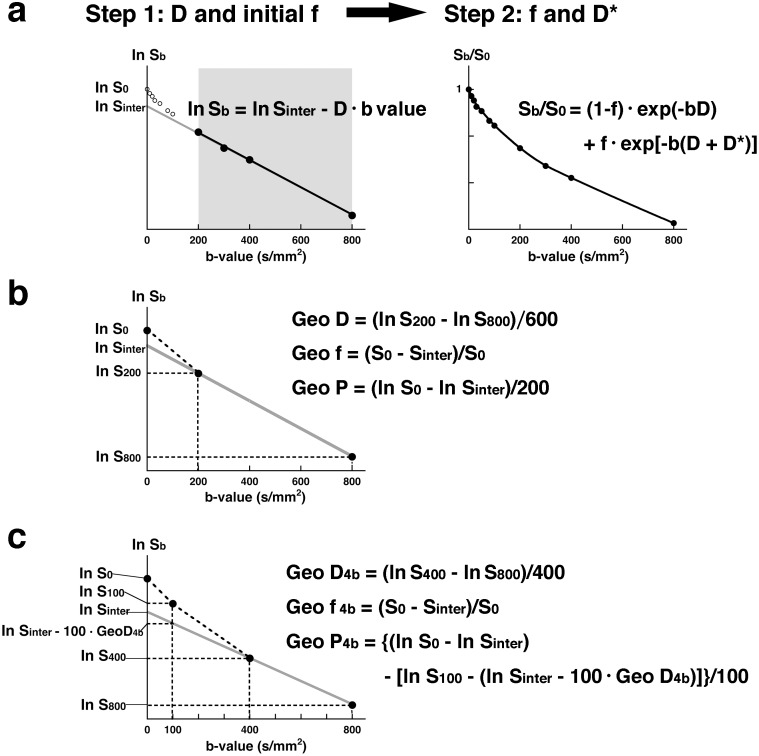
IVIM parameter determination by least-squares or geometric method. **a**, Least-squares method. Upper panel shows a representative signal decay curve obtained by using 11 b-values (0, 10, 20, 30, 50, 80, 100, 200, 300, 400, 800 s/mm^2^). At first step, D (Fit D) can be obtained by least-squares method using ln S_200_, ln S_300_, ln S_400_, and ln S_800_, and initial f value is calculated as 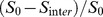, where S_inter_ is the interception of the logarithmic regression line obtained by using b-values of 200, 300, 400, and 800 s/mm^2^ with the y-axis. Right panel shows relationship between S_b_/S_0_ and varying b-values. Given D and initial f and D* values, f (Fit f) and D* (Fit D*) values can be obtained by least-squares method based on the equation: 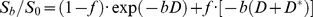. **b**, Geometric method. Graph shows geometric determination of IVIM parameters using 3 (0, 200, and 800 s/mm^2^) of the 11 b-values. D is calculated by the equation 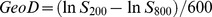. f is estimated by the equation 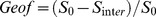, and P is estimated by the equation 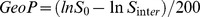
Geo P = (ln S_0_–In S_inter_)/200. **c**, Geometric method based on 4-b-value data. Graph shows geometric determination of IVIM parameters using 4 (0, 100, 400, and 800 s/mm^2^) of the 11 b-values. D is calculated by the equation 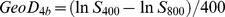, f is estimated by the equation 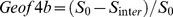, and P is estimated by the equation 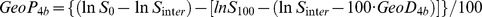.

The relationship between signal intensities and b-values based on the IVIM theory can be expressed using the following equation:
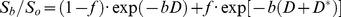
(1)where f is microvascular volume fraction, D is pure diffusion coefficient, and D* represents perfusion-related incoherent microcirculation [4]; S_0_ and S_b_ are signal intensities at b = 0 and b = 10, 20, 30, 50, 80, 100, 200, 300, 400, or 800 s/mm^2^, respectively. Using logarithmic plots (Fig. 1a), D (Fit D) can be obtained with a linear regression algorithm (the least-squares methods using b-values of 200, 300, 400, and 800 s/mm^2^). Given a D value, the initial f value was estimated as y-axis intersection of the linear regression (Fig. 1a). Then, the corresponding f (Fit f) and D* (Fit D*) values can be calculated using a nonlinear regression algorithm based on equation (1) (Fig. 1a). Fit f and Fit D* values were obtained after substituting initial f and D* values into the Levenberg-Marquardt algorithm [13], using SPSS software (Version 18.0, IBM incorporation). The initial values used for the least-squares method were as follows: f = −0.06–0.49 (average, 0.11±0.08); D* = 0.01 [5], [7]. The convergence criterion was 0.00000001.

### IVIM analysis based on geometric method

Separately, we analyzed signal decay curves by using the geometric method as described previously [6], [7], [9]. By using logarithmic plots, D can be estimated as a decline between b = 200–800 s/mm^2^, (ln S_200_–In S_800_)/600 (Fig. 1b). Given an estimated D value, we estimated the tissue perfusion by geometrically estimating the f as 1–S_inter_/S_0_ (S_inter_ is the interception of the logarithmic regression line obtained using b-values of 200 and 800 s/mm^2^ with the y-axis) (Fig. 1). On the other hand, perfusion property can be geometrically estimated by the formula as (ln S_0_–In S_inter_)/200. Fit D* reflects the vascular space only. However, the geometrically defined perfusion parameter is heavily weighted towards the perfusion in the extravascular space. Therefore, the geometrically perfusion parameter is fundamentally different from Fit D*. We introduced a perfusion parameter Geo P, which reflects and averages the vascular and extravascular spaces.

Separately, we determined IVIM parameters based on 4 b-value (b = 0, 100, 400, and 800 s/mm^2^) data according to the followings (Fig. 1c):
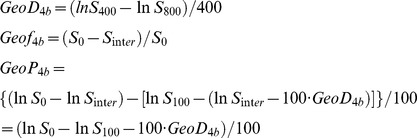



DW images in a DICOM format were converted to 2D color maps of geometrically determined f, D, and D* values by using the ImageJ software (NIH, http://rsweb.nih.gov/ij/index.html). We used an existing fit plug-in for ImageJ software. The color maps were generated purely for qualitative illustration and were not employed in the quantitative performance comparison of the least squares and geometrical methods for calculating IVIM parameters.

### Interobserver and intraobserver errors

Separate sets of DW MR images, including conventional T1- and T2-weighted, contrast-enhanced T1-weighted, and DW MR images, from 5 patients with head and neck tumors were analyzed independently by 3 separate radiologists with 17–20-year experience. The radiologists were asked to place an ROI onto each of DW MR images at b-values of 0, 200, and 800 s/mm^2^. One day after, the same radiologists were asked to repeat the same procedure with the same sets of DW MR images. Interobserver and intraobserver errors were assessed by calculating percent coefficient of variation (%CV) of IVIM parameters obtained from different ROIs placed on the same DW MR images.

### Statistics

Wilcoxon signed-rank test was used for the comparison of the IVIM parameters between the 2 techniques. Steel-Dwass test was used for the comparison of the IVIM parameters between the 3 different types of salivary gland tumors. Mann-Whitney U-test was used for the comparison of the IVIM parameters between lymphomas and SCCs. Cluster analysis was used to determine the best threshold for the IVIM criteria for discriminating between different tumor groups, where the best cutoff IVIM values were determined so that the values differentiated with the highest accuracy between different tumor groups that were categorized by Ward’s method using dendrogram. The statistical analyses were performed using SPSS (Version 18.0, IBM Corporation) and Excel Statistics 2012 (Version 1.00; SSRI).

## Results

### Errors in ROI placement

Interobserver and intraobserver errors of IVIM parameters were similar between Geo and Fit methods, except for intraobserver errors of D* values (Tables 2, S1, and S3).

**Table 2:** Inter- and intraobserver errors in measuring Geo and Fit IVIM parameters.

	IVIM parameters	%CV	
		Geo	Fit
Interobserver errors	D	0.5±0.2	0.5±0.3
	f	5.2±0.8	9.7±7.8
	P/D*	5.1±0.8	16.2±11.0
Intraobserver errors	D	0.9±0.9	1.0±0.9
	f	5.4±5.4	11.4±7.9
	P/D*	5.5±5.5^a^	19.7±8.6^a^

%CV, percent coefficient of variation; Geo, geometric measurement; Fit, least-squares method. P/D*, Geo P/Fit D*.

a, significant difference between Geo and Fit values (p = 0.0195, Wilcoxon signed-rank test).

### Computation-induced invalidity

IVIM imaging of 18 out of the 105 head and neck tumors resulted in invalid IVIM values from the whole ROI, including 4 tumors, in which f values were negative with geometric method; 3 tumors, in which initial f values were negative with least-squares method; and 17 tumors, in which obtained f or D* values were the same as the initial values with least-squares method. Consequently, IVIM parameters were not able to be determined in 14 tumors owing to computation-induced invalidity with the least-squares method alone, and 4 tumors owing to measurement errors with both methods.

### Differences in values of IVIM parameters between geometric and least-squares methods

**Figure 2:**
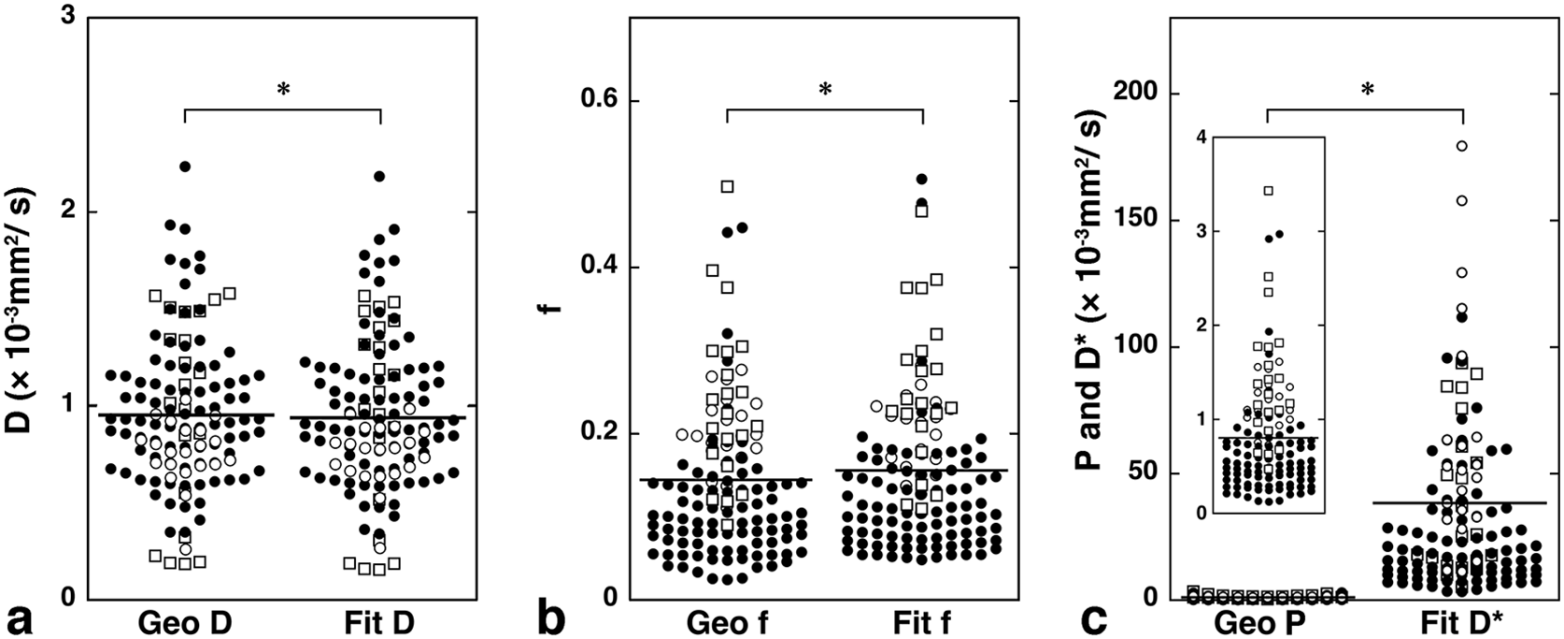
IVIM parameters of normal structures and tumors in the head and neck region. Plot graphs show D (Geo D), f (Geo f), and P (Geo P) values that were determined by geometric method; and D (Fit D), f (Fit f), and D* (Fit D*) values that were determined by least-squares method of normal structures (parotid glands, open circles; and masseter muscles, open squares) and head and neck tumors (closed circles). Broken white contours indicate tumor areas. Parotid gland: Geo D, Geo f and Geo P = 0.76±0.17×10^−3^ mm^2^/s, 0.20±0.04, and 1.12±0.27×10^−3^ mm^2^/s, respectively; and Fit D, Fit f, and Fit D* = 0.75±0.16×10^−3^ mm^2^/s, 0.20±0.05, and 62.96±46.78×10^−3^ mm^2^/s, respectively. Masseter muscle: Geo D, Geo f, and Geo P = 0.99±0.51×10^−3^ mm^2^/s, 0.24±0.10, and 1.41±0.71×10^−3^ mm^2^/s, respectively; and Fit D, Fit f, and Fit D* = 0.96±0.51×10^−3^ mm^2^/s, 0.25±0.10, and 40.50±30.13×10^−3^ mm^2^/s, respectively. Tumors: Geo D, Geo f, and Geo P = 1.00±0.38×10^−3^ mm^2^/s, 0.11±0.08, and 0.61±0.48×10^−3^ mm^2^/s, respectively; Fit D, Fit f, and Fit D* = 0.99±0.37×10^−3^ mm^2^/s, 0.12±0.08, and 24.14±21.15×10^−3^ mm^2^/s, respectively. Insert, Geo P distribution on a small scale. The values are the results of integrated signal intensities within the ROIs. *, p<0.001 (Wilcoxon signed-rank test).

D values determined by the geometric method (Geo D, 0.96±0.39×10^−3^ mm^2^/s) were significantly (p<0.001) greater than those determined by the least squares method (Fit D, 0.94±0.38×10^−3^ mm^2^/s) (Fig. 2, Tables S1 and S2). Geo f values (0.15±0.09) were significantly (p<0.001) smaller than Fit f valued (0.16±0.09). The differences were very small (2±4% for D values and −7±24% for f values) between 2 techniques.

### Differences in diagnostic abilities of IVIM parameters between geometric and least-squares methods

**Figure 3:**
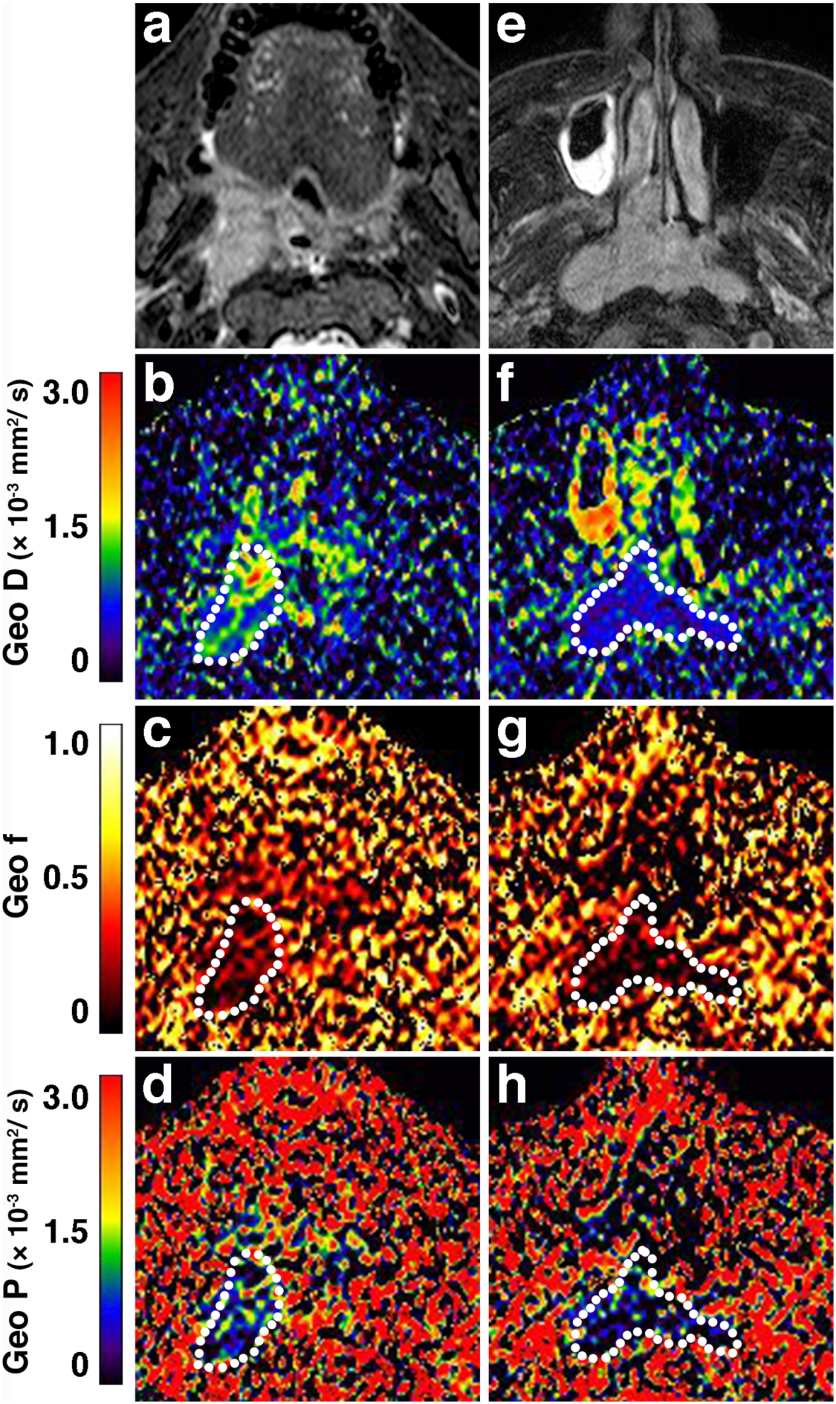
IVIM maps of SCC and lymphoma. **a–d**, Axial fat-suppressed T2-weighted MR image (**a**), and Geo D (**b**), Geo f (**c**), and Geo P (**d**) maps of 72-year-old man with SCC in oropharynx show tumor with homogeneous T2-signals and IVIM parameter values of Geo D, Geo f, and Geo P = 1.16×10^−3^ mm^2^/s, 0.14, and 0.76×10^−3^ mm^2^/s, respectively; and Fit D, Fit f, and Fit D* = 1.14×10^−3^ mm^2^/s, 0.18, and 8.50×10^−3^ mm^2^/s, respectively. **e–h**, Axial fat-suppressed T2-weighted MR image (e), and Geo D (f), Geo f (g), and Geo P (h) maps of 79-year-old man with lymphoma in nasopharynx show tumor with homogeneous T2 signals and IVIM parameter values of Geo D, Geo f, and Geo P = 0.59×10^−3^ mm^2^/s, 0.08, and 0.41×10^−3^ mm^2^/s, respectively; and Fit D, Fit f, and Fit D* = 0.60×10^−3^ mm^2^/s, 0.07, and 17.01×10^−3^ mm^2^/s, respectively. The values are the results of integrated signal intensities within the ROIs.

Given the significant differences in values of IVIM parameters between geometric and least squares methods, we next tested whether these differences would affect the diagnostic abilities of IVIM parameters in differentiating SCCs and lymphomas (Fig. 3, Tables 3, S1, and S4). We found that f and P/D* values were ineffective for differentiating the 2 types of malignant tumors, resulting in 59% accuracy with Geo f and Fit f values; and 59% and 50% accuracy with Geo P and Fit D*, respectively. However, D values differentiated between SCCs and lymphomas with diagnostic abilities of 71% sensitivity, 100% specificity, and 78% accuracy with Geo D; and 74% sensitivity, 100% specificity, and 80% accuracy with Fit D.

**Table 3:** IVIM parameters of SCCs and lymphomas.

IVIM parameter	SCC (n = 34)	Lymphoma (n = 12)
D (×10^−3 ^mm^2^/s)		
Geo	0.93±0.23^a^	0.63±0.16^a^
Fit	0.93±0.23^b^	0.62±0.15^b^
f		
Geo	0.13±0.10	0.09±0.04
Fit	0.14±0.10	0.10±0.03
P/D* (×10^−3^ mm^2^/s)		
Geo	0.74±0.65	0.49±0.23
Fit	27.11±22.06	28.52±15.01

IVIM, intravoxel incoherent motion; SCC, squamous cell carcinoma; Geo, IVIM parameters determined by geometric method; Fit, IVIM parameters determined by least squares method. P/D*, Geo P/Fit D*.

a, b significant differences (p = 0.0002, Mann-Whitney U test).

**Figure 4:**
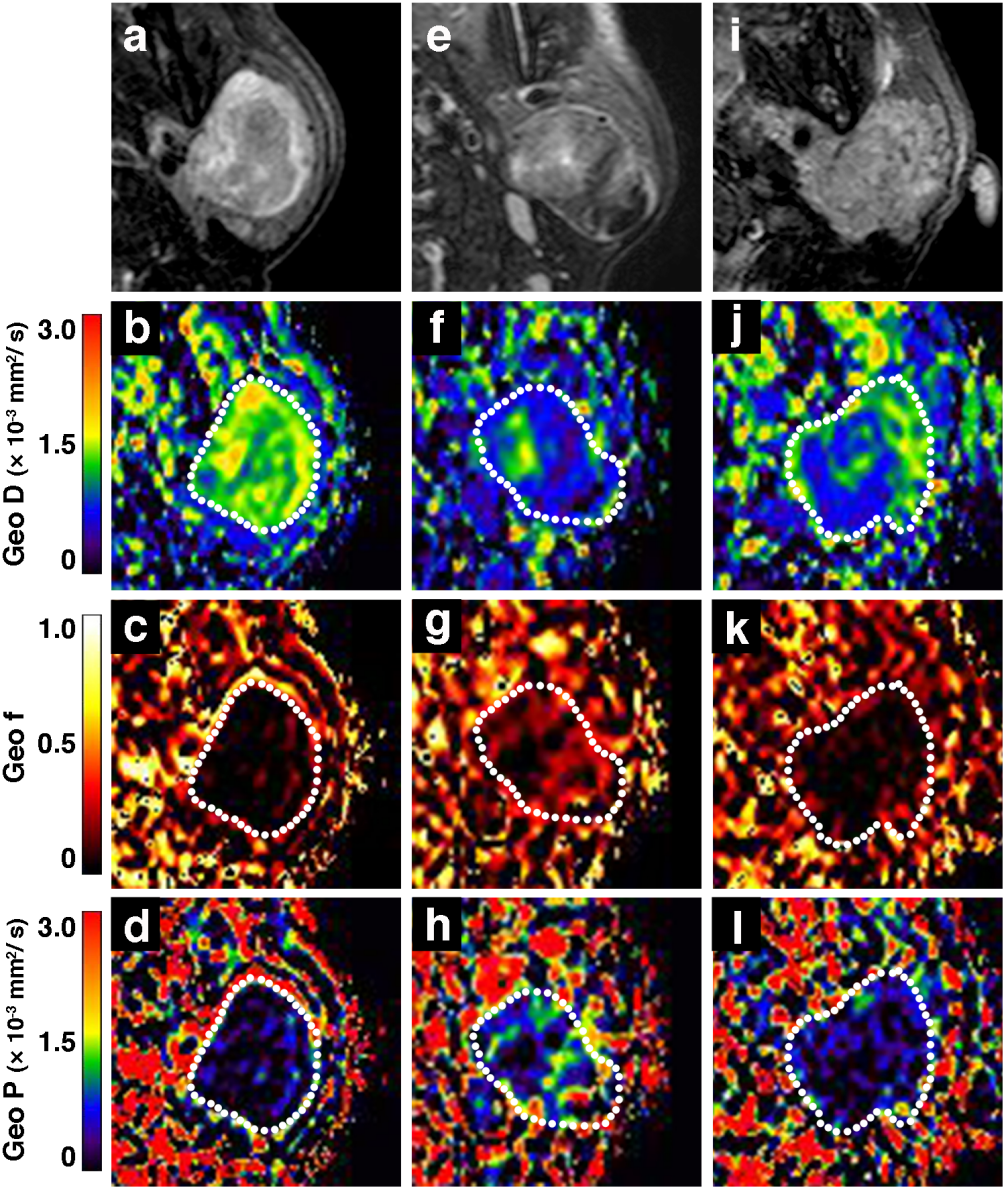
IVIM maps of benign and malignant salivary gland tumors. **a–d**, Axial fat-suppressed T2-weighted MR image (**a**), and Geo D (**b**), Geo f (**c**), and Geo P (**d**) maps of 67-year-old man with pleomorphic adenoma in left parotid gland show tumor with heterogeneous T2-signals and IVIM parameter values of Geo D, Geo f, and Geo P = 1.37×10^−3^ mm^2^/s, 0.02, and 0.12×10^−3^ mm^2^/s, respectively; and Fit D, Fit f, and Fit D* = 1.37×10^−3^ mm^2^/s, 0.05, and 4.23×10^−3^ mm^2^/s, respectively. Broken white contours indicate tumor areas. **e–h**, Axial fat-suppressed T2-weighted MR image (**e**), and Geo D (**f**), Geo f (**g**), and Geo P (**h**) maps of 65-year-old woman with Warthin tumor in left parotid gland show tumor with heterogeneous T2-signals and IVIM parameter values of Geo D, Geo f, and Geo P = 0.87×10^−3^ mm^2^/s, 0.14, and 0.75×10^−3^ mm^2^/s, respectively; and Fit D, Fit f, and Fit D* = 0.84×10^−3^ mm^2^/s, 0.16, and 23.32×10^−3^ mm^2^/s, respectively. Broken white contours indicate tumor areas. **i–l**, Axial fat-suppressed T2-weighted MR image (**i**), and Geo D (**j**), Geo f (**k**), and Geo P (**l**) maps of 59-year-old woman with carcinoma ex. Pleomorphic adenoma in left parotid gland show tumor with heterogeneous T2-signals and IVIM parameter values of Geo D, Geo f, and Geo P = 0.89×10^−3^ mm^2^/s, 0.04, and 0.20×10^−3^ mm^2^/s, respectively; and Fit D, Fit f, and Fit D* = 0.88×10^−3^ mm^2^/s, 0.05, and 10.00×10^−3^ mm^2^/s, respectively. Broken white contours indicate tumor areas. The values are the results of integrated signal intensities within the ROIs.

**Figure 5:**
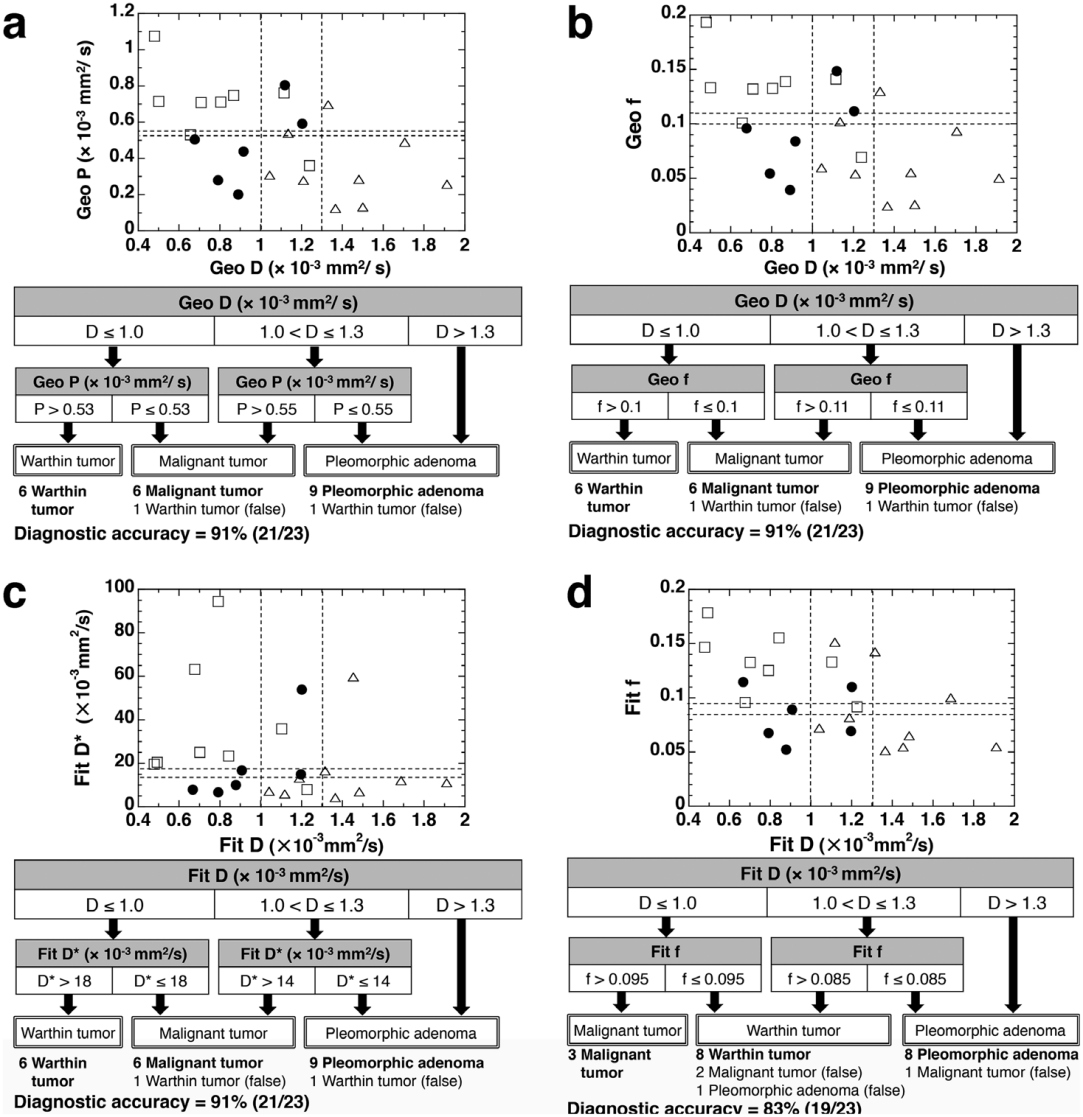
Stepwise differentiation between pleomorphic adenomas, Warthin tumors, and malignant salivary gland tumors using D, f, and D* or P values that were determined by geometric (Geo) or least-squares (Fit) method. Plot graphs show 2D distributions of Geo P and GeoD (**a**), Geo f and Geo D (**b**), Fit D* and Fit D (**C**), or Fit f and Fit D (**d**). Open triangles, open squares, and closed circles indicate pleomorphic adenomas, Warthin tumors, and malignant salivary gland tumors, respectively. In combinations of Geo D and Geo P (a), Geo D and Geo f, or Fit D and Fit D*, stepwise approach diagnosed 21 of 23 salivary gland tumors correctly; in these approaches, the same Warthin tumor was incorrectly diagnosed as a malignant salivary gland tumor owing to having a large Geo D ( = 1.11×10^−3^ mm^2^/s) or Fit D values ( = 1.11×10^−3^ mm^2^/s); or incorrectly diagnosed as a pleomorphic adenoma owing to having a large Geo D ( = 1.24×10^−3^ mm^2^/s) or Fit D ( = 1.23×10^−3^ mm^2^/s) and small Geo P ( = 0.36×10^−3^ mm^2^/s) or Fit D* ( = 7.90×10^−3^ mm^2^/s) values. The diagnostic accuracy with stepwise approach using Fit D and Fit f was lower than that using the corresponding geometric parameters (**b, d**). Diagnostic accuracy was provided for the respective classifications at the bottom of each diagram.

Although significant differences in the IVIM values were found between the different types of salivary gland tumors (pleomorphic adenomas, Warthin tumors, and malignant salivary gland tumors), any single use of the parameters was ineffective in discriminating between the different tumor types (Fig. 4, Tables 4, S1, and S5). Therefore, we attempted to discriminate between the 3 different tumor types by using a stepwise approach with combined uses of the 3 IVIM parameters that were determined by the geometric or the least-squares methods (Fig. 5. Tables S1 and S5). The stepwise differentiation using Geo D and Geo P (Fig. 5a), Geo D and Geo f (Fig. 5b), or Fit D and Fit D* (Fig. 5c) differentiated 21 (91%) of the 23 salivary gland tumors correctly; consequently, the same 2 Warthin tumors were incorrectly diagnosed as malignant tumor or pleomorphic adenoma. However, a stepwise approach using Fit D and Fit f differentiated the salivary gland tumors less effectively; 3 malignant tumors were incorrectly diagnosed as Warthin tumors or pleomorphic adenoma; and 1 pleomorphic adenoma as Warthin tumor (Fig. 5d).

**Table 4:** IVIM parameters of pleomorphic adenomas, Warthin tumors, and malignant SG tumors.

IVIM parameter	Pleomophic adenoma (n = 9)	Warthin tumor (n = 8)	Malignant SG tumor (n = 6)
D (×10^−3^ mm^2^/s)			
Geo	1.41±0.28^a,b^	0.80±0.27^a^	0.93±0.20^b^
Fit	1.40±0.28^a,b^	0.79±0.27^a^	0.94±0.22^b^
f			
Geo	0.07±0.04^c^	0.13±0.04^c^	0.09±0.04
Fit	0.09±0.04	0.13±0.03^d^	0.08±0.03^d^
P/D* (×10^−3^ mm^2^/s)			
Geo	0.35±0.19^e^	0.70±0.20^e^	0.47±0.22
Fit	15.16±17.15^f^	36.23±28.61^f^	18.38±17.84

IVIM, intravoxel incoherent motion; SG, salivary gland; Geo, IVIM parameters determined by geometrical method; Fit, IVIM parameters determined by least squares method. P/D*, Geo P/Fit D*.

a–f significant differences (p<0.05, Steel-Dwass test).

**Figure 6:**
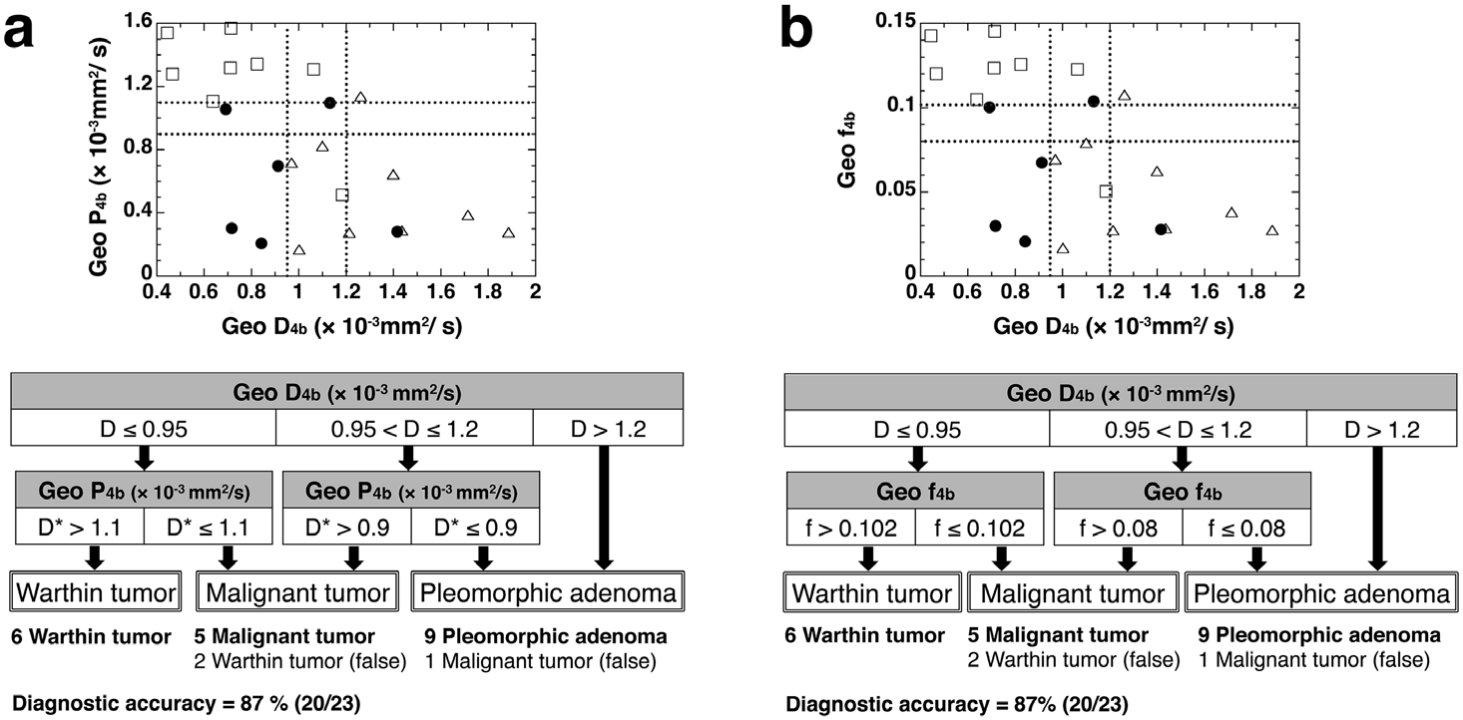
Stepwise differentiation between pleomorphic adenomas, Warthin tumors, and malignant salivary gland tumors using D, f, and D* or P values that were determined by geometric (Geo) method using 4 b-values (0, 100, 400, and 800 s/mm^2^). Plot graphs show 2D distributions of Geo P and GeoD (**a**), or Geo f and Geo D (**b**). Open triangles, open squares, and closed circles indicate pleomorphic adenomas, Warthin tumors, and malignant salivary gland tumors, respectively. Diagnostic accuracy was provided for the respective classifications at the bottom of each diagram.

Lastly, we tested whether the use of 4 b-values (0, 100, 400, and 800 s/mm^2^) could significantly influence the IVIM parameter levels and their diagnostic abilities compared with the use of 3 b-values (0, 200, and 800 s/mm^2^). We found that Geo D and Geo f values of salivary gland tumors determined by the 3 b- or 4 b-values were not significantly different (Tables 5, S1, and S5). Furthermore, the use of 4 b-values resulted in less effective differentiation of salivary gland tumors compared with the IVIM parameters that were determined using 3 b-values, and 3 tumors were incorrectly diagnosed (Fig. 6, Tables S1 and S5).

**Table 5:** IVIM parameters of 23 salivary gland tumors that were determined by least squares, 3b-geometrical, or 4b-geometrical methods.

IVIM parameters	Fit	3b-Geo	4b-Geo
D (×10^−3^ mm^2^/s)	1.07±0.37	1.07±0.37	1.03±0.38
f	0.10±0.04	0.09±0.04	0.08±0.04
P/D* (×10^−3^ mm^2^/s)	23.3±23.1^a,b^	0.50±0.25^a^	0.80±0.47^b^

IVIM, intravoxel incoherent motion; Fit, least squares method; 3b-Geo, geometric method using 3 b-values; 4b-Geo, geometric method using 4 b-values. P/D*, Geo P/Fit D*.

a, b significant differences (p<0.05) (Steel-Dwass test).

## Discussion

The present results showed that levels of IVIM parameters that were determined by geometric method were significantly different from those determined by least-squares method. However, differences in levels of D and f values were very small between the 2 methods, and diagnostic abilities of geometrically determined IVIM parameters were equivalent to those of IVIM parameters determined by least-squares method in differentiating between lymphomas and SCCs, and between different types of salivary gland tumors (pleomorphic adenomas, Warthin tumors, and malignant salivary gland tumors). Considering cumbersome procedures with least-squares method, the simple geometric IVIM assessment could be an alternative to least-squares method in the clinics.

By using a limited number of b-values, IVIM imaging has the advantage of achieving DW MR images that have better quality and of examining broader areas of head and neck region in a single scan compared with IVIM imaging using more b-values; for example, IVIM imaging using 11 b-values requires 1 min 53 s for obtaining 10 DW image slices per patient; on the other hand, IVIM imaging using 3 b-values requires 26 s for obtaining the same number of DW images per patient. The 3 b-value IVIM technique abandoned the idea of using low b-value (<100 s/mm^2^) DWI for a fine analysis of the different vascular compartments with different sizes of vessels [14]–[16]. Perfusion contributes to signal decays in DWI in a biexponential mode for b-values in very low range (0–200 s/mm^2^) [3], [14], [15]. Indeed, the significant differences in D, f, and D* values between the geometric and least-squares methods may be owing to the use of the upper limit of this b-value range (200 s/mm^2^) for assessing the perfusion-related and pure molecular diffusion parameters separately in the present study. However, the use of 4 b-values did not improve the diagnostic abilities in differentiating the different tumor types. These results suggest that the use of 3 b-values (0, 200, and 800 s/mm^2^) is clinically feasible for assessing perfusion and diffusion of head and neck tumors in routine examinations.

The present study showed that computation-induced invalidity occurred less frequently with the geometric method compared with the least-squares one. However, these results do not necessarily ensure the better performance of the implied IVIM technique. Basically, the least-squares method has better performance than the geometric method in terms of predicting lesional perfusion and diffusion characteristics with less artifacts and higher signal-to-noise ratios compared with the simplified technique. For example, some perfusion property may be lost during the simplified IVIM parameter calculation with limited numbers of small (<200 s/mm^2^) b-values. Furthermore, many of the computation-induced errors with the least-squares technique could be avoided through the use of appropriate scan setting and/or b-values.

The 3 b-value geometric assessment of IVIM parameters was slightly less effective in the diagnosis of salivary gland tumors compared with a previous study, which achieved 100% accuracy [5]. The difference in study cohort may be a possible reason for the difference in diagnostic accuracy. In the present study, stepwise approaches using Fit D and Fit D* or Geo D and Geo P diagnosed 21 of the 23 salivary gland tumors correctly (91% accuracy). In both the combinations of Fit and Geo IVIM parameters, the 2 same Warthin tumors were incorrectly diagnosed as malignant or pleomorphic adenoma (Fig. 5). However, diagnostic accuracy with stepwise approaches using Fit D and Fit f values was lower than that using Geo D and Geo f values (19/23 = 83% vs 21/23 = 91%), implying advantages of geometric method for the clinical use in diagnosing salivary gland tumors.

A major limitation of this study was the small patient cohort. Different or additional cutoff points might be required for effective discrimination in a larger patient cohort that is comprised of increased numbers of tumors within each tumor type and broader types of head and neck tumors. For example, the present study cohort of salivary gland tumor did not include oncocytoma, which histologically mimics Warthin tumor or malignant salivary gland tumors such as acinic cell carcinoma and clear cell carcinoma [17]. The retrospective nature, including the exclusion of patients with severe susceptibility artifacts from the study cohort, also limit the value of this study. In addition, the benefit of using the simplified IVIM technique may largely depends on disease types. Furthermore, the perfusion-related parameter Geo P defined by the simplified IVIM technique is fundamentally different from the conventional one (Fit D*). For example, in some cases where the diffusion property is important for diagnosing tumors/diseases, the simplified IVIM technique may be beneficial; however, in other cases where the perfusion assessment is essential for diagnosing tumors/disease, the simplified technique will provide perfusion parameters that are greatly different from those obtained with the least squares technique using multiple b-values and may thus mislead the diagnosis.

Another limitation of the present study may reside in ROI placement errors. We found that the interobserver and intraobserver errors were relatively small. However, there were substantial overlaps in IVIM parameters between different tumor types, and thus a small change in the value due to ROI placement may lead to a different result in tumor categorization based on IVIM imaging. In addition, distortion of tumor area due to susceptibility and motion artifacts may be critical factors against precise IVIM parameter measurements.

## Conclusion

In this study, we showed that the IVIM parameters determined by geometric method were significantly different from those determined by conventional least-squares method. Nonetheless, both yielded very similar results in terms of differential diagnosis of major types of head and neck tumors, including SCCs, lymphomas, and salivary gland tumors. Therefore, we concluded that geometric determination of IVIM parameters could be an alternative to least-squares methods in the diagnosis of head and neck tumors.

## Supporting Information

Table S1 All numerical data for Figs. 2, 5 and 6, and Tables 2, 3, 4 and 5 are summarized. Signal intensities relative to varying b-values (0–800 s/mm^2^) are shown for each of benign (n = 26) and malignant (n = 61) head and neck tumors.
(DOCX)

Table S2 IVIM parameters (Geo D, Geo f, Geo P, Fit D, Fit f, and Fit D*) are shown for each of benign (n = 26) and malignant (n = 61) head and neck tumors.
(DOCX)

Table S3 IVIM parameters (Geo D, Geo f, Geo P, Fit D, Fit f, and Fit D*) determined 5 times (#1–#5) by 3 observers (1–3) are shown for 5 head and neck tumors (Cases 1–5).
(DOCX)

Table S4 IVIM parameters (Geo D, Geo f, Geo P, Fit D, Fit f, and Fit D*) for lymphomas (n = 12), primary SCCs (n = 23), and SCC nodes (n = 11) are shown.
(DOCX)

Table S5 IVIM parameters determined by least squares method (Fit D, Fit f, and Fit D*), geometrical method using 3 b-values (Geo D3b, Geo f3b, and Geo P3b), or geometrical method using 4 b-values (Geo D4b, Geo f4b, Geo P4b) for benign (n = 17) and malignant (n = 6) salivary gland tumors are shown.
(DOCX)
