# Supplementary material for: Simple and Reliable Determination of Intravoxel Incoherent Motion Parameters for the Differential Diagnosis of Head and Neck Tumors
Source: PLoS One. 2014 Nov 17;9(11):e112866. doi: 10.1371/journal.pone.0112866 (PMC4234537; doi:10.1371/journal.pone.0112866)
Supplement: Table S1 — All numerical data for Figs. 2, 5 and 6, and Tables 2, 3, 4 and 5 are summarized. Signal intensities relative to varying b-values (0–800 s/mm2) are shown for each of benign (n = 26) and malignant (n = 61) head and neck tumors. (DOCX) [file pone.0112866.s001.docx]

| **Table S1. Data for all figures and tables** | | | | | | | | | | | | | |
| --- | --- | --- | --- | --- | --- | --- | --- | --- | --- | --- | --- | --- | --- |
| Pathology | | | Signal intensity/b-value (s/mm^2^) | | | | | | | | | | |
|  |  |  | 0 | 10 | 20 | 30 | 50 | 80 | 100 | 200 | 300 | 400 | 800 |
| Adopted cases (n = 87) | | |  |  |  |  |  |  |  |  |  |  |  |
| Benign lesions (n = 26) | | |  |  |  |  |  |  |  |  |  |  |  |
|  | Adenomatous goiter | | 190.108 | 166.944 | 173.052 | 150.77 | 151.192 | 146.366 | 134.519 | 104.607 | 78.896 | 58.582 | 27.363 |
|  | Ameloblastoma | | 206.345 | 198.034 | 187.891 | 185.202 | 173.387 | 167.622 | 153.345 | 125.504 | 101.605 | 85.084 | 43.277 |
|  | Ameloblastoma | | 132.198 | 128.891 | 124.799 | 123.316 | 119.087 | 112.813 | 110.5 | 95.249 | 84.327 | 74.405 | 47.539 |
|  | Angiomyoma | | 186.253 | 179.138 | 177.361 | 174.892 | 171.886 | 159.639 | 154.576 | 127.831 | 112.804 | 85.167 | 48.07 |
|  | Keratocystic odontogenic tumor | | 112.045 | 111.56 | 110.873 | 109.733 | 107.09 | 103.651 | 101.124 | 92.536 | 84.347 | 76.682 | 56.515 |
|  | Myofibroma | | 93.897 | 90.304 | 87.234 | 86.4 | 81.935 | 81.358 | 78.07 | 68.899 | 60.662 | 55.699 | 33.603 |
|  | Odontogenic fibroma | | 153.845 | 141.945 | 133.974 | 129.547 | 124.788 | 117.697 | 113.516 | 97.232 | 85.819 | 76.63 | 46.291 |
|  | Odontogenic fibroma | | 66.55 | 64.594 | 64.486 | 61.142 | 57.7 | 55.203 | 52.631 | 44.067 | 36.133 | 30.289 | 15.367 |
|  | Papilloma | | 224.213 | 227.541 | 210.918 | 213.743 | 207.127 | 183.34 | 181.866 | 138.187 | 104.134 | 79.194 | 43.321 |
|  | Pleomorphic adenoma | | 128.162 | 123.597 | 123.981 | 121.004 | 116.481 | 110.591 | 107.867 | 90.03 | 78.173 | 65.724 | 37.013 |
|  | Pleomorphic adenoma | | 172.795 | 169.009 | 164.803 | 162.376 | 156.425 | 149.743 | 145.962 | 123.65 | 108.671 | 92.246 | 62.581 |
|  | Pleomorphic adenoma | | 146.873 | 140.405 | 136.622 | 135.13 | 130.324 | 126.324 | 119.729 | 105.984 | 85.139 | 75.39 | 43.08 |
|  | Pleomorphic adenoma | | 93.528 | 92.203 | 88.832 | 88.442 | 85.746 | 79.589 | 75.797 | 60.284 | 49.173 | 43 | 21.655 |
|  | Pleomorphic adenoma | | 104.923 | 103.186 | 100.923 | 98.764 | 96.552 | 93.666 | 93.342 | 80.059 | 71.98 | 63.851 | 42.782 |
|  | Pleomorphic adenoma | | 125.52 | 122.082 | 119.455 | 119.51 | 115.312 | 111.538 | 108.156 | 93.16 | 82.186 | 66.666 | 41.038 |
|  | Pleomorphic adenoma | | 141.787 | 134.602 | 130.638 | 127.37 | 122.687 | 116.011 | 111.529 | 94.529 | 81.082 | 70.467 | 42.539 |
|  | Pleomorphic adenoma | | 126.869 | 122.838 | 119.818 | 119.718 | 115.155 | 110.464 | 104.67 | 94.22 | 81.399 | 70.79 | 45.595 |
|  | Pleomorphic adenoma | | 125.436 | 124.556 | 121.495 | 118.595 | 113.623 | 106.264 | 101.03 | 81.267 | 67.03 | 54.843 | 25.79 |
|  | Warthin tumor | | 66.319 | 62.843 | 60.827 | 60.195 | 59.33 | 58.53 | 55.697 | 52.005 | 47.762 | 46.4 | 38.503 |
|  | Warthin tumor | | 92.96 | 87.128 | 85.646 | 83.869 | 83.953 | 78.535 | 73.98 | 73.327 | 70.832 | 65.828 | 49.485 |
|  | Warthin tumor | | 97.184 | 88.916 | 87.284 | 86.453 | 83.884 | 80.133 | 76.664 | 66.79 | 58.887 | 52.355 | 34.222 |
|  | Warthin tumor | | 92.285 | 83.839 | 81.211 | 80.668 | 78.914 | 77.301 | 74.301 | 68.155 | 61.459 | 58.478 | 42.061 |
|  | Warthin tumor | | 79.287 | 72.947 | 70.947 | 70.719 | 70.374 | 67.69 | 65.018 | 58.099 | 56.713 | 52.012 | 43.55 |
|  | Warthin tumor | | 96.98 | 89.857 | 88.55 | 85.724 | 84.888 | 79.685 | 79.168 | 70.213 | 62.63 | 55.46 | 41.732 |
|  | Warthin tumor | | 85.8 | 80.289 | 79.137 | 77.954 | 75.499 | 73.06 | 72.063 | 64.619 | 59.817 | 54.492 | 42.231 |
|  | Warthin tumor | | 176.627 | 172.105 | 168.732 | 166.32 | 161.14 | 153.963 | 149.034 | 128.287 | 111.621 | 97.938 | 61 |
| Malignant lesions (n = 61) | | |  |  |  |  |  |  |  |  |  |  |  |
|  | Acinic cell carcinoma | | 86.429 | 85.806 | 84.497 | 82.558 | 81.648 | 78.313 | 78.055 | 69.761 | 64.939 | 57.79 | 43.397 |
|  | Adenoid cystic carcinoma | | 142.705 | 142.395 | 140.884 | 139.07 | 135.736 | 129.29 | 119.822 | 112.667 | 103.403 | 98.953 | 75.054 |
|  | Carcinoma ex. pleomorphic adenoma | | 105.186 | 104.155 | 103.168 | 101.655 | 101.027 | 96.831 | 94.703 | 84.569 | 76.186 | 69.451 | 49.586 |
|  | Carcinoma ex. pleomorphic adenoma | | 86.406 | 83.362 | 83.248 | 82.682 | 79.335 | 73.343 | 72.918 | 58.821 | 58.485 | 52.971 | 30.07 |
|  | Dedifferentiated ca. | | 123.28 | 114.896 | 112.241 | 108.635 | 104.792 | 99.495 | 98.659 | 86.096 | 76.549 | 65.771 | 41.831 |
|  | Lymohoma | | 112.237 | 107.235 | 106.692 | 104.961 | 103.635 | 101.836 | 96.242 | 90.967 | 79.719 | 77.755 | 54.98 |
|  | Lymohoma | | 116.317 | 113.569 | 112.676 | 110.439 | 109.71 | 104.955 | 102.204 | 95.18 | 91.839 | 83.889 | 66.767 |
|  | Lymohoma | | 159.685 | 149.426 | 148.833 | 139.019 | 136.796 | 135.722 | 134.667 | 123.481 | 109.204 | 92.907 | 73.815 |
|  | Lymohoma | | 114.944 | 110.591 | 107.558 | 105.982 | 102.581 | 99.698 | 98.66 | 88.44 | 80.8 | 74.284 | 56.148 |
|  | Lymohoma | | 119.586 | 116.476 | 114.677 | 113.722 | 112.054 | 106.633 | 104.499 | 93.923 | 86.094 | 81.457 | 59.041 |
|  | Lymohoma | | 75.638 | 74.538 | 72.726 | 72.654 | 67.897 | 66.295 | 65.722 | 60.184 | 56.449 | 53.115 | 41.701 |
|  | Lymohoma | | 119.257 | 111.929 | 110.986 | 110.486 | 106.329 | 105.1 | 101.971 | 96.814 | 92.486 | 74.7 | 67.729 |
|  | Lymohoma | | 120.209 | 113.434 | 107.214 | 104.391 | 102.318 | 101.134 | 97.799 | 90.951 | 84.24 | 75.557 | 62.704 |
|  | Lymohoma | | 142.586 | 141.486 | 138.97 | 136.014 | 135.629 | 128.314 | 127.029 | 115.643 | 112.743 | 100 | 80.657 |
|  | Lymohoma | | 76.924 | 74.741 | 74 | 72.59 | 71.533 | 68.448 | 66.7 | 65.287 | 59.316 | 58.31 | 45.257 |
|  | Lymohoma | | 86.015 | 76.805 | 78.752 | 74.969 | 75.347 | 75.446 | 72.616 | 64.92 | 64.7 | 57.985 | 52.591 |
|  | Lymohoma | | 77.743 | 76.004 | 74.913 | 73.111 | 72.763 | 72.055 | 69.466 | 67.498 | 64.458 | 61.593 | 54.672 |
|  | Lymph node metastasis  fadenocarcinoma | | 81.073 | 78.885 | 76.372 | 76.437 | 74.922 | 72.639 | 70.163 | 62.997 | 57.687 | 50.448 | 37.028 |
|  | Lymph node metastasis  adenocarcinoma | | 97.56 | 95.321 | 92.956 | 90.818 | 88.075 | 84.057 | 80.415 | 68.95 | 61.522 | 55.05 | 40.006 |
|  | Lymph node metastasis  adenoid cystic carcinoma | | 67.151 | 65.101 | 63.721 | 61.911 | 61.547 | 58.33 | 55.346 | 51.251 | 42.765 | 39.464 | 29.324 |
|  | Lymph node metastasis  dedifferentiated carcinoma | | 130.317 | 126.446 | 124.703 | 121.851 | 118.614 | 112.267 | 109.881 | 99.267 | 85.356 | 80.812 | 52.119 |
|  | Lymph node metastasis  papillary carcinoma | | 120.713 | 116.033 | 111.488 | 111.167 | 108.943 | 104.488 | 74.402 | 71.215 | 70.906 | 69.856 | 46.636 |
|  | Lymph node metastasis  papillary carcinoma | | 155.873 | 153.537 | 149.002 | 145.039 | 138.691 | 128.129 | 127.984 | 108.859 | 84.825 | 73.32 | 44.317 |
|  | Malignant melanoma | | 119.76 | 115.66 | 113.743 | 113.511 | 110.279 | 105.055 | 101.733 | 89.673 | 79.802 | 70.693 | 47.973 |
|  | Nasopharyngeal carcinoma | | 100.867 | 94.663 | 93.084 | 92.74 | 91.019 | 87.437 | 85.631 | 76.854 | 69.27 | 64.38 | 52.863 |
|  | Papillary carcinoma | | 104.034 | 99.594 | 98.91 | 98.325 | 95.632 | 95.406 | 87.073 | 76.756 | 73.62 | 62.128 | 47.803 |
|  | Salivary duct carcinoma | | 57.134 | 54.714 | 53.839 | 53.324 | 52.572 | 50.038 | 48.639 | 43.574 | 39.199 | 36.225 | 25.152 |
|  | SCC (squamous cell carcinoma, primary) | | 100.064 | 107.909 | 88.938 | 83.161 | 68.944 | 59.343 | 70.126 | 47.957 | 39.576 | 39.308 | 31.373 |
|  | SCC | | 76.688 | 75.948 | 73.266 | 71.148 | 69.425 | 64.858 | 64.219 | 57.956 | 51.329 | 44.488 | 30.989 |
|  | SCC | | 92.989 | 90.249 | 89.729 | 88.026 | 85.356 | 83.472 | 82.266 | 77.046 | 71.207 | 65.983 | 51.634 |
|  | SCC | | 82.023 | 72.847 | 76.883 | 73.506 | 65.506 | 64.068 | 64.277 | 60.006 | 56.906 | 55.185 | 43.312 |
|  | SCC | | 94.215 | 89.565 | 87.834 | 86.744 | 82.063 | 80.901 | 76.713 | 69.659 | 59.946 | 58.865 | 40.426 |
|  | SCC | | 164.775 | 154.871 | 148.9 | 147.217 | 142.488 | 137.17 | 133.489 | 116.191 | 100.522 | 88.722 | 57.955 |
|  | SCC | | 121.93 | 117.446 | 116.375 | 114.012 | 111.844 | 109.469 | 103.736 | 96.924 | 87.15 | 80.073 | 57.386 |
|  | SCC | | 157.115 | 150.772 | 148.569 | 146.184 | 142.372 | 137.953 | 135.419 | 126.175 | 114.495 | 108.181 | 83.884 |
|  | SCC | | 74.753 | 68.301 | 66.768 | 65.528 | 64.949 | 60.007 | 58.591 | 52.603 | 48.381 | 43.241 | 30.735 |
|  | SCC | | 85.217 | 82.814 | 81.186 | 78.907 | 77.326 | 73.76 | 72.016 | 63.76 | 57.279 | 52.915 | 36.372 |
|  | SCC | | 83.538 | 79.849 | 78.231 | 77.109 | 73.504 | 70.613 | 69.916 | 60.474 | 52.437 | 47.349 | 31.277 |
|  | SCC | | 112.01 | 107.538 | 107.459 | 103.917 | 100.949 | 100.229 | 100.182 | 83.796 | 73.803 | 59.815 | 38.914 |
|  | SCC | | 86.001 | 80.392 | 77.643 | 76.229 | 74.555 | 70.748 | 68.616 | 61.39 | 53.469 | 47.164 | 32.289 |
|  | SCC | | 128.876 | 120.194 | 118.641 | 114.829 | 109.175 | 102.382 | 96.171 | 86.544 | 85.195 | 79.378 | 49.433 |
|  | SCC | | 119.381 | 115.972 | 112.881 | 109.622 | 104.497 | 101.012 | 96.382 | 81.415 | 71.79 | 60.555 | 40.704 |
|  | SCC | | 101.096 | 93.724 | 93.072 | 92.862 | 91.399 | 84.275 | 64.668 | 51.914 | 51.778 | 46.322 | 40.501 |
|  | SCC | | 105.125 | 98.633 | 92.675 | 92.317 | 89.075 | 83.182 | 70.583 | 68.083 | 64.525 | 39.925 | 30.475 |
|  | SCC | | 112.058 | 109.462 | 107.492 | 107.228 | 105.118 | 100.847 | 99.025 | 89.997 | 82.349 | 76.915 | 56.063 |
|  | SCC | | 108.425 | 106.062 | 103.938 | 103.669 | 96.381 | 92.312 | 90.488 | 76.1 | 71.844 | 65.794 | 38.794 |
|  | SCC | | 94.857 | 89.933 | 88.893 | 88.891 | 86.114 | 80.767 | 80.851 | 72.278 | 65.628 | 58.908 | 41.233 |
|  | SCC | | 73.787 | 68.385 | 68.277 | 66.986 | 66.941 | 64.804 | 62.769 | 55.615 | 42.811 | 40.192 | 30.85 |
|  | SCC | | 118.246 | 114.966 | 111.96 | 108.046 | 104.337 | 103.349 | 101.931 | 92.257 | 83.88 | 75.291 | 51.863 |
|  | SCC | | 81.026 | 79.078 | 78.676 | 75.691 | 72.331 | 71.156 | 64.32 | 55.276 | 49.977 | 44.842 | 30.779 |
|  | SCC node | | 81.628 | 78.324 | 76.341 | 74.531 | 74.427 | 72.698 | 69.751 | 61.841 | 57.709 | 55.282 | 45.885 |
|  | SCC node | | 104.301 | 101.068 | 98.586 | 96.6 | 92.386 | 88.769 | 86.602 | 73.006 | 64.304 | 58.245 | 33.271 |
|  | SCC node | | 124.972 | 124.431 | 121.34 | 119.791 | 113.977 | 107.418 | 108.987 | 94.219 | 83.675 | 71.113 | 45.602 |
|  | SCC node | | 101.851 | 90.5 | 83.05 | 71.347 | 69.315 | 65.677 | 64.698 | 63.117 | 59.312 | 58.734 | 32.946 |
|  | SCC node | | 65.664 | 62.875 | 62.855 | 61.579 | 57.546 | 56.559 | 57.375 | 51.125 | 48.625 | 46.303 | 35.237 |
|  | SCC node | | 71.24 | 63.689 | 60.93 | 58.826 | 57.083 | 54.885 | 52.741 | 48.207 | 43.71 | 36.935 | 25.832 |
|  | SCC node | | 45.123 | 45.081 | 43.851 | 42.581 | 41.981 | 39.721 | 39.144 | 35.712 | 31.933 | 29.014 | 20.516 |
|  | SCC node | | 95.964 | 91.592 | 90.979 | 88.815 | 85.879 | 83.372 | 79.454 | 71.06 | 63.834 | 57.038 | 38.601 |
|  | SCC node | | 121.297 | 93.841 | 89.792 | 88.908 | 85.786 | 81.34 | 79.565 | 70.834 | 61.602 | 53.108 | 39.033 |
|  | SCC node | | 103.238 | 101.764 | 99.231 | 96.245 | 93.521 | 93.346 | 92.532 | 84.989 | 78.359 | 71.955 | 54.67 |
|  | SCC node | | 89.803 | 89.629 | 85.813 | 83.463 | 81.832 | 81.803 | 79.117 | 70.298 | 65.654 | 60.962 | 42.565 |
|  |  | |  |  |  |  |  |  |  |  |  |  |  |
| Excluded cases (n = 18) | | |  |  |  |  |  |  |  |  |  |  |  |
| Benign lesions (n = 9) | | |  |  |  |  |  |  |  |  |  |  |  |
|  | Hemangioma | | 178.615 | 177.008 | 174.546 | 174.397 | 171.492 | 165.162 | 147.662 | 138.192 | 115.9 | 106.177 | 66.262 |
|  | Keratocystic odontogenic tumor | | 175.956 | 175.515 | 173.578 | 171.134 | 168 | 161.047 | 156.878 | 139.016 | 123.314 | 111.388 | 77.236 |
|  | Odontogenic myxoma | | 144.722 | 143.571 | 143.101 | 140.909 | 133.679 | 125.034 | 118.593 | 94.894 | 76.662 | 60.093 | 23.252 |
|  | Pleomorphic adenoma | | 159.351 | 159.972 | 156.725 | 154.074 | 147.04 | 140.394 | 134.984 | 109.697 | 90.32 | 73.287 | 33.898 |
|  | Pleomorphic adenoma | | 90.374 | 88.877 | 88.213 | 86.09 | 86.045 | 81.968 | 81.019 | 74.71 | 60.581 | 46.632 | 31.69 |
|  | Pleomorphic adenoma | | 167.802 | 165.297 | 164.211 | 162.345 | 158.111 | 149.808 | 146.029 | 126.502 | 107.668 | 93.962 | 55.246 |
|  | Pleomorphic adenoma | | 93.996 | 90.031 | 89.76 | 89.105 | 84.511 | 82.786 | 78.668 | 66.162 | 57.668 | 54.611 | 26.332 |
|  | Pleomorphic adenoma | | 170.109 | 166.743 | 166.439 | 163.94 | 160.69 | 153.263 | 149.347 | 129.32 | 113.916 | 98.316 | 61.363 |
|  | Pleomorphic adenoma | | 92.838 | 89.035 | 88.521 | 88.035 | 87.546 | 85.94 | 82.949 | 71.743 | 60.121 | 57.362 | 34.308 |
| Malignant lesions (n = 9) | | |  |  |  |  |  |  |  |  |  |  |  |
|  | | Adenocarcinoma | 59.801 | 57.705 | 58.446 | 58.706 | 57.533 | 55.696 | 54.377 | 48.936 | 44.743 | 40.631 | 30.348 |
|  | | Ameloblastic carcinoma | 151.961 | 145.855 | 144.559 | 142.879 | 139.449 | 131.325 | 127.084 | 105.558 | 89.306 | 77.502 | 46.807 |
|  | | Lymph node metastasis  papillary carcinoma | 74.807 | 73.158 | 72.982 | 70.982 | 70.596 | 70.596 | 69.789 | 61.561 | 54.456 | 52.228 | 41.86 |
|  | | Lymphoma | 64.654 | 62.138 | 62.524 | 62.81 | 62.244 | 62.375 | 60.822 | 55.766 | 55.58 | 53.148 | 42.05 |
|  | | Lymphoma | 137.152 | 136.14 | 135.733 | 135.207 | 132.3 | 129.201 | 123.874 | 118.571 | 101.168 | 91.162 | 67.228 |
|  | | Neuroendocrine carcinoma | 75.58 | 67.416 | 66.294 | 63.234 | 62.39 | 61.745 | 60.94 | 54.494 | 53.472 | 50.905 | 33.684 |
|  | | SCC | 92.071 | 91.361 | 91 | 88.408 | 86.71 | 82.867 | 75.831 | 68.839 | 58.616 | 61.051 | 43.62 |
|  | | SCC | 69.867 | 64.81 | 65.169 | 66 | 63.01 | 61.634 | 60.438 | 57.402 | 53.156 | 45.194 | 31.276 |
|  | | SCC node | 95.964 | 94.082 | 93.578 | 91.974 | 90.036 | 85.414 | 80.184 | 68.772 | 62.246 | 57.639 | 39.266 |
