# Supplementary material for: Simple and Reliable Determination of Intravoxel Incoherent Motion Parameters for the Differential Diagnosis of Head and Neck Tumors
Source: PLoS One. 2014 Nov 17;9(11):e112866. doi: 10.1371/journal.pone.0112866 (PMC4234537; doi:10.1371/journal.pone.0112866)
Supplement: Table S3 — IVIM parameters (Geo D, Geo f, Geo P, Fit D, Fit f, and Fit D*) determined 5 times (#1–#5) by 3 observers (1–3) are shown for 5 head and neck tumors (Cases 1–5). (DOCX) [file pone.0112866.s003.docx]

| **Table S3.** Data for Table 2. | | | | | | | | |
| --- | --- | --- | --- | --- | --- | --- | --- | --- |
|  |  |  |  |  |  |  |  |  |
| **Case** | **Measurement** | | **IVIM parameters** | | | | | |
|  |  |  | **Geo D　　　　　(×10^-3^mm^2^/s)** | **Geo f** | **Geo P**  **(×10^-3^mm^2^/s)** | **Fit D**  **(×10^-3^mm^2^/s)** | **Fit f** | **Fit D***  **(×10^-3^mm^2^/s)** |
| Case 1 | Observer 1 | #1 | 1.348 | 0.040 | 0.196 | 1.354 | 0.051 | 2.265 |
|  |  | #2 | 1.375 | 0.026 | 0.128 | 1.374 | 0.070 | 1.913 |
|  |  | #3 | 1.373 | 0.025 | 0.125 | 1.372 | 0.088 | 1.902 |
|  |  | #4 | 1.371 | 0.027 | 0.132 | 1.371 | 0.051 | 1.960 |
|  |  | #5 | 1.369 | 0.026 | 0.127 | 1.368 | 0.050 | 1.921 |
|  | Observer 2 | #1 | 1.372 | 0.029 | 0.145 | 1.371 | 0.042 | 1.063 |
|  |  | #2 | 1.373 | 0.030 | 0.147 | 1.372 | 0.050 | 2.088 |
|  |  | #3 | 1.371 | 0.028 | 0.139 | 1.370 | 0.031 | 2.008 |
|  |  | #4 | 1.375 | 0.030 | 0.147 | 1.374 | 0.042 | 2.075 |
|  |  | #5 | 1.375 | 0.029 | 0.144 | 1.374 | 0.031 | 2.020 |
|  | Observer 3 | #1 | 1.373 | 0.033 | 0.161 | 1.372 | 0.046 | 2.196 |
|  |  | #2 | 1.372 | 0.032 | 0.159 | 1.371 | 0.052 | 2.182 |
|  |  | #3 | 1.375 | 0.031 | 0.154 | 1.374 | 0.044 | 2.117 |
|  |  | #4 | 1.375 | 0.031 | 0.154 | 1.374 | 0.044 | 2.117 |
|  |  | #5 | 1.369 | 0.031 | 0.152 | 1.369 | 0.033 | 1.120 |
|  |  |  |  |  |  |  |  |  |
| Case 2 | Observer 1 | #1 | 0.890 | 0.040 | 0.201 | 0.878 | 0.043 | 3.136 |
|  |  | #2 | 0.954 | 0.048 | 0.246 | 0.941 | 0.054 | 2.997 |
|  |  | #3 | 0.958 | 0.052 | 0.264 | 0.946 | 0.054 | 2.101 |
|  |  | #4 | 0.961 | 0.051 | 0.257 | 0.949 | 0.053 | 3.114 |
|  |  | #5 | 0.961 | 0.051 | 0.257 | 0.949 | 0.053 | 3.988 |
|  | Observer 2 | #1 | 0.961 | 0.051 | 0.258 | 0.950 | 0.054 | 1.000 |
|  |  | #2 | 0.949 | 0.050 | 0.252 | 0.937 | 0.054 | 1.010 |
|  |  | #3 | 0.950 | 0.050 | 0.253 | 0.938 | 0.054 | 1.981 |
|  |  | #4 | 0.949 | 0.049 | 0.248 | 0.937 | 0.053 | 1.100 |
|  |  | #5 | 0.952 | 0.049 | 0.250 | 0.939 | 0.053 | 1.900 |
|  | Observer 3 | #1 | 0.961 | 0.051 | 0.261 | 0.949 | 0.054 | 3.102 |
|  |  | #2 | 0.962 | 0.054 | 0.276 | 0.959 | 0.049 | 2.710 |
|  |  | #3 | 0.942 | 0.059 | 0.301 | 0.930 | 0.058 | 2.991 |
|  |  | #4 | 0.957 | 0.056 | 0.284 | 0.946 | 0.055 | 1.502 |
|  |  | #5 | 0.963 | 0.057 | 0.290 | 0.952 | 0.053 | 3.180 |
|  |  |  |  |  |  |  |  |  |
| Case 3 | Observer 1 | #1 | 1.239 | 0.071 | 0.360 | 1.227 | 0.051 | 4.230 |
|  |  | #2 | 1.238 | 0.073 | 0.367 | 1.226 | 0.092 | 8.334 |
|  |  | #3 | 1.237 | 0.072 | 0.365 | 1.225 | 0.092 | 8.307 |
|  |  | #4 | 1.244 | 0.075 | 0.377 | 1.232 | 0.094 | 8.775 |
|  |  | #5 | 1.254 | 0.073 | 0.369 | 1.242 | 0.094 | 8.266 |
|  | Observer 2 | #1 | 1.254 | 0.074 | 0.373 | 1.242 | 0.094 | 8.420 |
|  |  | #2 | 1.260 | 0.074 | 0.372 | 1.248 | 0.093 | 8.503 |
|  |  | #3 | 1.262 | 0.075 | 0.379 | 1.250 | 0.093 | 8.934 |
|  |  | #4 | 1.254 | 0.076 | 0.381 | 1.242 | 0.093 | 9.089 |
|  |  | #5 | 1.241 | 0.078 | 0.392 | 1.229 | 0.098 | 8.510 |
|  | Observer 3 | #1 | 1.241 | 0.080 | 0.401 | 1.228 | 0.096 | 10.085 |
|  |  | #2 | 1.245 | 0.080 | 0.401 | 1.232 | 0.095 | 10.272 |
|  |  | #3 | 1.181 | 0.092 | 0.467 | 1.164 | 0.118 | 8.638 |
|  |  | #4 | 1.248 | 0.079 | 0.398 | 1.235 | 0.095 | 16.043 |
|  |  | #5 | 1.249 | 0.078 | 0.394 | 1.237 | 0.094 | 9.795 |
|  |  |  |  |  |  |  |  |  |
| Case 4 | Observer 1 | #1 | 1.135 | 0.104 | 0.538 | 1.089 | 0.171 | 7.267 |
|  |  | #2 | 1.148 | 0.103 | 0.532 | 1.131 | 0.148 | 6.115 |
|  |  | #3 | 1.166 | 0.103 | 0.542 | 1.147 | 0.151 | 6.366 |
|  |  | #4 | 1.164 | 0.104 | 0.535 | 1.145 | 0.144 | 7.226 |
|  |  | #5 | 1.157 | 0.104 | 0.537 | 1.137 | 0.147 | 6.816 |
|  | Observer 2 | #1 | 1.168 | 0.104 | 0.533 | 1.148 | 0.146 | 6.998 |
|  |  | #2 | 1.159 | 0.105 | 0.538 | 1.140 | 0.148 | 6.702 |
|  |  | #3 | 1.151 | 0.106 | 0.547 | 1.132 | 0.151 | 6.497 |
|  |  | #4 | 1.157 | 0.104 | 0.535 | 1.139 | 0.148 | 6.523 |
|  |  | #5 | 1.150 | 0.105 | 0.540 | 1.131 | 0.150 | 6.311 |
|  | Observer 3 | #1 | 1.170 | 0.102 | 0.525 | 1.152 | 0.145 | 6.628 |
|  |  | #2 | 1.164 | 0.103 | 0.532 | 1.145 | 0.147 | 6.442 |
|  |  | #3 | 1.152 | 0.106 | 0.548 | 1.134 | 0.153 | 6.127 |
|  |  | #4 | 1.157 | 0.104 | 0.533 | 1.138 | 0.144 | 7.149 |
|  |  | #5 | 1.155 | 0.105 | 0.537 | 1.136 | 0.145 | 7.042 |
|  |  |  |  |  |  |  |  |  |
| Case 5 | Observer 1 | #1 | 1.118 | 0.154 | 0.805 | 1.197 | 0.055 | 28.727 |
|  |  | #2 | 1.098 | 0.155 | 0.808 | 1.169 | 0.062 | 28.921 |
|  |  | #3 | 1.097 | 0.158 | 0.825 | 1.168 | 0.067 | 27.194 |
|  |  | #4 | 1.098 | 0.159 | 0.831 | 1.170 | 0.067 | 27.849 |
|  |  | #5 | 1.100 | 0.159 | 0.831 | 1.174 | 0.063 | 30.057 |
|  | Observer 2 | #1 | 1.095 | 0.166 | 0.866 | 1.170 | 0.069 | 29.007 |
|  |  | #2 | 1.099 | 0.163 | 0.853 | 1.174 | 0.067 | 27.305 |
|  |  | #3 | 1.103 | 0.160 | 0.833 | 1.175 | 0.067 | 27.985 |
|  |  | #4 | 1.102 | 0.163 | 0.849 | 1.177 | 0.065 | 29.255 |
|  |  | #5 | 1.102 | 0.164 | 0.854 | 1.178 | 0.065 | 28.726 |
|  | Observer 3 | #1 | 1.102 | 0.162 | 0.842 | 1.178 | 0.063 | 29.520 |
|  |  | #2 | 1.100 | 0.162 | 0.845 | 1.174 | 0.066 | 28.339 |
|  |  | #3 | 1.099 | 0.159 | 0.827 | 1.172 | 0.063 | 30.634 |
|  |  | #4 | 1.100 | 0.160 | 0.838 | 1.174 | 0.064 | 29.326 |
|  |  | #5 | 1.098 | 0.159 | 0.828 | 1.171 | 0.065 | 28.286 |
