# Supplementary material for: Simple and Reliable Determination of Intravoxel Incoherent Motion Parameters for the Differential Diagnosis of Head and Neck Tumors
Source: PLoS One. 2014 Nov 17;9(11):e112866. doi: 10.1371/journal.pone.0112866 (PMC4234537; doi:10.1371/journal.pone.0112866)
Supplement: Table S4 — IVIM parameters (Geo D, Geo f, Geo P, Fit D, Fit f, and Fit D*) for lymphomas (n = 12), primary SCCs (n = 23), and SCC nodes (n = 11) are shown. (DOCX) [file pone.0112866.s004.docx]

| **Table S4.**  Data for Table 3 | | | | | | |
| --- | --- | --- | --- | --- | --- | --- |
|  |  |  |  |  |  |  |
| **Pathology** | **IVIM parameters** | | | | | |
|  | **Geo D　　　　　　　　(×10^-3^mm^2^/s)** | **Geo f** | **Geo P**  **(×10^-3^mm^2^/s)** | **Fit D**  **(×10^-3^mm^2^/s)** | **Fit f** | **Fit D***  **(×10^-3^mm^2^/s)** |
| Lymphoma | 0.839 | 0.041 | 0.211 | 0.812 | 0.055 | 53.297 |
| Lymphoma | 0.591 | 0.079 | 0.412 | 0.604 | 0.072 | 17.014 |
| Lymphoma | 0.620 | 0.144 | 0.775 | 0.604 | 0.151 | 42.108 |
| Lymphoma | 0.601 | 0.085 | 0.447 | 0.618 | 0.073 | 12.301 |
| Lymphoma | 0.858 | 0.082 | 0.428 | 0.822 | 0.112 | 53.950 |
| Lymphoma | 0.757 | 0.105 | 0.553 | 0.746 | 0.112 | 20.017 |
| Lymphoma | 0.774 | 0.083 | 0.434 | 0.769 | 0.087 | 11.364 |
| Lymphoma | 0.611 | 0.041 | 0.209 | 0.591 | 0.062 | 34.923 |
| Lymphoma | 0.351 | 0.190 | 1.056 | 0.365 | 0.169 | 28.555 |
| Lymphoma | 0.351 | 0.069 | 0.355 | 0.342 | 0.079 | 21.541 |
| Lymphoma | 0.611 | 0.101 | 0.531 | 0.609 | 0.105 | 15.512 |
| Lymphoma | 0.595 | 0.086 | 0.447 | 0.587 | 0.106 | 31.701 |
| SCC (squamous cell carcinoma, primary) | 0.707 | 0.448 | 2.970 | 0.627 | 0.506 | 11.665 |
| SCC | 1.043 | 0.069 | 0.357 | 1.026 | 0.095 | 15.727 |
| SCC | 0.667 | 0.053 | 0.273 | 0.657 | 0.064 | 27.009 |
| SCC | 0.543 | 0.184 | 1.019 | 0.547 | 0.182 | 34.755 |
| SCC | 0.907 | 0.114 | 0.603 | 0.873 | 0.134 | 18.484 |
| SCC | 1.159 | 0.111 | 0.587 | 1.137 | 0.114 | 39.554 |
| SCC | 0.874 | 0.053 | 0.274 | 0.860 | 0.065 | 27.802 |
| SCC | 0.680 | 0.080 | 0.416 | 0.661 | 0.095 | 24.558 |
| SCC | 0.896 | 0.158 | 0.861 | 0.897 | 0.149 | 39.383 |
| SCC | 0.936 | 0.098 | 0.515 | 0.927 | 0.104 | 14.499 |
| SCC | 1.099 | 0.098 | 0.517 | 1.076 | 0.117 | 15.684 |
| SCC | 1.278 | 0.034 | 0.173 | 1.269 | 0.083 | 3.354 |
| SCC | 1.071 | 0.116 | 0.615 | 1.046 | 0.128 | 36.484 |
| SCC | 0.933 | 0.191 | 1.058 | 0.992 | 0.145 | 33.164 |
| SCC | 1.155 | 0.141 | 0.758 | 1.141 | 0.177 | 8.503 |
| SCC | 0.414 | 0.442 | 2.919 | 0.433 | 0.477 | 7.486 |
| SCC | 1.340 | 0.153 | 0.832 | 1.356 | 0.182 | 23.051 |
| SCC | 0.789 | 0.060 | 0.307 | 0.783 | 0.065 | 12.927 |
| SCC | 1.123 | 0.121 | 0.647 | 1.165 | 0.077 | 17.857 |
| SCC | 0.935 | 0.081 | 0.424 | 0.932 | 0.083 | 27.628 |
| SCC | 0.982 | 0.083 | 0.431 | 0.868 | 0.226 | 5.546 |
| SCC | 0.960 | 0.055 | 0.281 | 0.959 | 0.060 | 59.035 |
| SCC | 0.976 | 0.171 | 0.936 | 0.972 | 0.197 | 7.265 |
| SCC node | 0.497 | 0.163 | 0.891 | 0.484 | 0.181 | 10.051 |
| SCC node | 1.310 | 0.090 | 0.474 | 1.317 | 0.075 | 21.243 |
| SCC node | 1.209 | 0.040 | 0.203 | 1.206 | 0.055 | 9.729 |
| SCC node | 1.084 | 0.230 | 1.309 | 1.123 | 0.231 | 111.800 |
| SCC node | 0.620 | 0.119 | 0.631 | 0.630 | 0.103 | 25.334 |
| SCC node | 1.040 | 0.167 | 0.913 | 1.036 | 0.172 | 71.595 |
| SCC node | 0.924 | 0.048 | 0.246 | 0.909 | 0.068 | 12.705 |
| SCC node | 1.017 | 0.092 | 0.485 | 1.012 | 0.097 | 18.608 |
| SCC node | 0.993 | 0.288 | 1.696 | 0.958 | 0.175 | 59.031 |
| SCC node | 0.735 | 0.046 | 0.237 | 0.728 | 0.052 | 43.707 |
| SCC node | 0.836 | 0.075 | 0.388 | 0.848 | 0.055 | 26.652 |
