# Supplementary material for: Simple and Reliable Determination of Intravoxel Incoherent Motion Parameters for the Differential Diagnosis of Head and Neck Tumors
Source: PLoS One. 2014 Nov 17;9(11):e112866. doi: 10.1371/journal.pone.0112866 (PMC4234537; doi:10.1371/journal.pone.0112866)
Supplement: Table S5 — IVIM parameters determined by least squares method (Fit D, Fit f, and Fit D*), geometrical method using 3 b-values (Geo D3b, Geo f3b, and Geo P3b), or geometrical method using 4 b-values (Geo D4b, Geo f4b, Geo P4b) for benign (n = 17) and malignant (n = 6) salivary gland tumors are shown. (DOCX) [file pone.0112866.s005.docx]

| **Table S5.**  Data for Table 4, Table 5, Fig. 5 and Fig. 6 | | | | | | | | | | |
| --- | --- | --- | --- | --- | --- | --- | --- | --- | --- | --- |
|  |  |  |  |  |  |  |  |  |  |  |
| **Pathology** | | **IVIM parameters** | | | | | | | | |
|  |  | **Geo D3b (×10^-3^mm^2^/s)** | **Geo f3b** | **Geo P3b (×10^-3^mm^2^/s)** | **Fit D (×10^-3^mm^2^/s)** | **Fit f** | **Fit D* (×10^-3^mm^2^/s)** | **Geo D4b (×10^-3^mm^2^/s)** | **Geo f4b** | **Geo P4b (×10^-3^mm^2^/s)** |
| Benign salivary gland tumors (n=17) | |  |  |  |  |  |  |  |  |  |
|  | Pleomorphic adenoma | 1.481 | 0.055 | 0.284 | 1.483 | 0.065 | 6.859 | 1.435 | 0.028 | 0.288 |
|  | Pleomorphic adenoma | 1.135 | 0.102 | 0.538 | 1.118 | 0.151 | 5.854 | 0.970 | 0.069 | 0.718 |
|  | Pleomorphic adenoma | 1.500 | 0.026 | 0.131 | 1.454 | 0.055 | 59.668 | 1.399 | 0.062 | 0.644 |
|  | Pleomorphic adenoma | 1.706 | 0.093 | 0.490 | 1.687 | 0.100 | 12.000 | 1.715 | 0.038 | 0.387 |
|  | Pleomorphic adenoma | 1.044 | 0.060 | 0.308 | 1.041 | 0.072 | 7.151 | 1.001 | 0.017 | 0.168 |
|  | Pleomorphic adenoma | 1.366 | 0.025 | 0.124 | 1.366 | 0.051 | 4.230 | 1.213 | 0.027 | 0.276 |
|  | Pleomorphic adenoma | 1.331 | 0.130 | 0.696 | 1.315 | 0.142 | 16.502 | 1.262 | 0.108 | 1.139 |
|  | Pleomorphic adenoma | 1.210 | 0.054 | 0.278 | 1.189 | 0.082 | 13.153 | 1.100 | 0.079 | 0.824 |
|  | Pleomorphic adenoma | 1.913 | 0.050 | 0.257 | 1.911 | 0.055 | 11.000 | 1.886 | 0.027 | 0.278 |
|  | Warthin tumor | 0.501 | 0.133 | 0.715 | 0.478 | 0.147 | 19.586 | 0.466 | 0.120 | 1.279 |
|  | Warthin tumor | 0.655 | 0.101 | 0.531 | 0.677 | 0.096 | 63.204 | 0.713 | 0.145 | 1.570 |
|  | Warthin tumor | 1.114 | 0.141 | 0.761 | 1.103 | 0.133 | 35.901 | 1.063 | 0.123 | 1.309 |
|  | Warthin tumor | 0.804 | 0.133 | 0.711 | 0.792 | 0.125 | 94.419 | 0.824 | 0.126 | 1.344 |
|  | Warthin tumor | 0.480 | 0.193 | 1.074 | 0.493 | 0.178 | 20.454 | 0.444 | 0.143 | 1.540 |
|  | Warthin tumor | 0.867 | 0.139 | 0.748 | 0.843 | 0.155 | 23.322 | 0.711 | 0.124 | 1.318 |
|  | Warthin tumor | 0.709 | 0.132 | 0.709 | 0.702 | 0.133 | 25.015 | 0.637 | 0.105 | 1.108 |
|  | Warthin tumor | 1.239 | 0.069 | 0.360 | 1.227 | 0.092 | 7.948 | 1.184 | 0.050 | 0.515 |
| Malignant salivary gland tumors (n=6) | |  |  |  |  |  |  |  |  |  |
|  | Adenoid cystic carcinoma | 0.677 | 0.096 | 0.505 | 0.667 | 0.115 | 7.921 | 0.691 | 0.100 | 1.057 |
|  | Acinic cell carcinoma | 0.791 | 0.054 | 0.280 | 0.792 | 0.068 | 6.734 | 0.716 | 0.030 | 0.303 |
|  | Carcinoma ex. pleomorphic adenoma | 0.890 | 0.039 | 0.201 | 0.878 | 0.052 | 10.000 | 0.842 | 0.021 | 0.208 |
|  | Carcinoma ex. pleomorphic adenoma | 1.118 | 0.149 | 0.805 | 1.197 | 0.069 | 14.982 | 1.416 | 0.028 | 0.282 |
|  | Dedifferentiated carcinoma | 1.203 | 0.112 | 0.592 | 1.201 | 0.110 | 53.903 | 1.131 | 0.104 | 1.097 |
|  | Salivary duct carcinoma | 0.916 | 0.084 | 0.439 | 0.907 | 0.089 | 16.767 | 0.912 | 0.067 | 0.698 |
